# Genome Editing in Root and Tuber Crop Development in Sub‐Saharan Africa

**DOI:** 10.1002/pei3.70158

**Published:** 2026-05-08

**Authors:** Himanshu Saini, Rohit Kumar, Manjeet, Srinu Banothu, Suram Likhita Reddy, Rajesh Gudihalli Munivenkatappa, Sri Sai Siddartha Naik, Roop Kumar, Rahul Pradhan

**Affiliations:** ^1^ School of Applied Natural Science Adama Science and Technology University Adama Ethiopia; ^2^ School of Agriculture Dev Bhoomi Uttarakhand University Dehradun Uttarakhand India; ^3^ Faculty of Agricultural Sciences GLA University Mathura Uttar Pradesh India; ^4^ Faculty of Agricultural Sciences Shree Guru Gobind Singh Tricentenary University Gurugram Haryana India; ^5^ School of Agriculture SR University Warangal Telangana India; ^6^ Department of Agronomy Marwadi University Rajkot Gujrat India; ^7^ Department of Crop Management Kumaraguru Institute of Agriculture Erode Tamil Nadu India; ^8^ Department of Agronomy PJTAU Agricultural College Warangal Telangana India; ^9^ School of Agriculture Lovely Professional University Phagwara Punjab India; ^10^ Silviculture and Forest Management Division Institute of Wood Science and Technology (ICFRE‐IWST) Bengaluru India

**Keywords:** climate change resilience, CRISPR/Cas9, crop improvement, precision genome editing, root and tuber crops

## Abstract

Precision genome editing, particularly using Clustered Regularly Interspaced Short Palindromic Repeats (CRISPR)/CRISPR‐associated protein 9 (Cas9), is advancing crop improvement by enabling targeted and efficient genetic modifications. Root and tuber crops such as potato, cassava, sweet potato, and yam are vital for global food and nutritional security but remain highly vulnerable to climate change, pests, diseases, and limited genetic diversity. Genome editing technologies facilitate the development of improved traits, including enhanced disease resistance, tolerance to abiotic stress, improved nutritional quality, and extended shelf life. This review synthesizes recent advances in genome editing for root and tuber crops across global production systems, including illustrative examples from Sub‐Saharan Africa, where active genome editing initiatives are being implemented. It further examines key technical constraints, such as low efficiency of plant transformation and regeneration, and highlights regulatory challenges arising from differing policy frameworks across countries. Emerging solutions are discussed, including genotype‐independent editing strategies and DNA‐free approaches that avoid the integration of foreign genetic material. Addressing these challenges will be critical for developing resilient and sustainable food systems. Unlike previous reviews, this study integrates mechanistic insights with cross‐crop synthesis and proposes next‐generation genome editing strategies for engineering complex traits in polyploid root and tuber crops.

## 1 Introduction

Root and tuber crops including potato, cassava, sweet potato, and yam anchor food and nutrition security for hundreds of millions of people, yet conventional breeding has struggled with their clonal propagation, high heterozygosity, and frequent polyploidy. Over the last decade, CRISPR‐based genome editing has helped address several of these bottlenecks, enabling trait gains in disease resistance, product quality, and stress tolerance while preserving elite backgrounds. CRISPR‐based genome editing overcomes key constraints of clonal propagation and polyploidy in root and tuber crops by enabling direct modification of elite genotypes without the need for recombination‐based breeding. Beyond nuclease knockouts, precision editors are now demonstrably useful in root and tuber crops. Cytosine base editors tailored for potato achieve efficient C → T conversion across multiple alleles in protoplast systems, opening routes to predictable amino‐acid substitutions without double‐strand breaks. Prime editing has also been shown to function in tetraploid potato at lower efficiencies than classical CRISPR but with the advantage of specifying exact nucleotide changes supporting the feasibility of allele‐level surgery in polyploids (Westberg et al. 2023; Perroud et al. 2022).

Trait‐level case studies are accumulating. In potato, editing susceptibility genes such as *StDMR6‐1* confers robust resistance to late blight. Multi‐year field evaluations demonstrate durable protection without detectable yield or quality penalties. Notably, edited lines also exhibit additional resistance to early blight and common scab, representing an unusually broad spectrum of disease resistance for a single gene modification (Kieu et al. 2021; Karlsson et al. 2024). In cassava, editing host translation initiation factors (*nCBP/eIF4E* family) reduces cassava brown streak disease by disrupting the interaction between viral VPg proteins and host eIF4E complexes, which are required for cap‐dependent viral RNA translation. Loss or modification of these host factors prevents efficient viral genome translation and replication, thereby conferring resistance while direct knockouts of cyanogenic glucoside pathway genes (e.g., *CYP79D1/D2*) lower root cyanide potential by blocking the initial step of linamarin biosynthesis, where these cytochrome P450 enzymes catalyze the conversion of valine into oxime intermediates. Disruption of this pathway prevents downstream formation of cyanogenic glucosides, thereby improving food safety. Sweet potato, despite its hexaploidy, is now increasingly amenable to genome editing; targeted modification of starch biosynthesis genes such as *IbGBSSI* and *IbSBEII* has demonstrated improvements in starch composition and quality traits (Wang et al. 2019). In addition, RNA‐targeting approaches using CRISPR/Cas13 have enabled resistance to viral pathogens such as sweet potato virus disease through degradation of viral transcripts (Yu et al. 2022). Yam, which has long been considered recalcitrant to genetic transformation, has recently seen the establishment of efficient CRISPR/Cas9 mutagenesis systems, as demonstrated by targeted editing of *DrPDS*, providing a foundation for future trait improvement (Syombua et al. 2021). Broader advances in genome editing of vegetatively propagated crops have been summarized in recent reviews (Tripathi et al. 2024).

**FIGURE 1:**
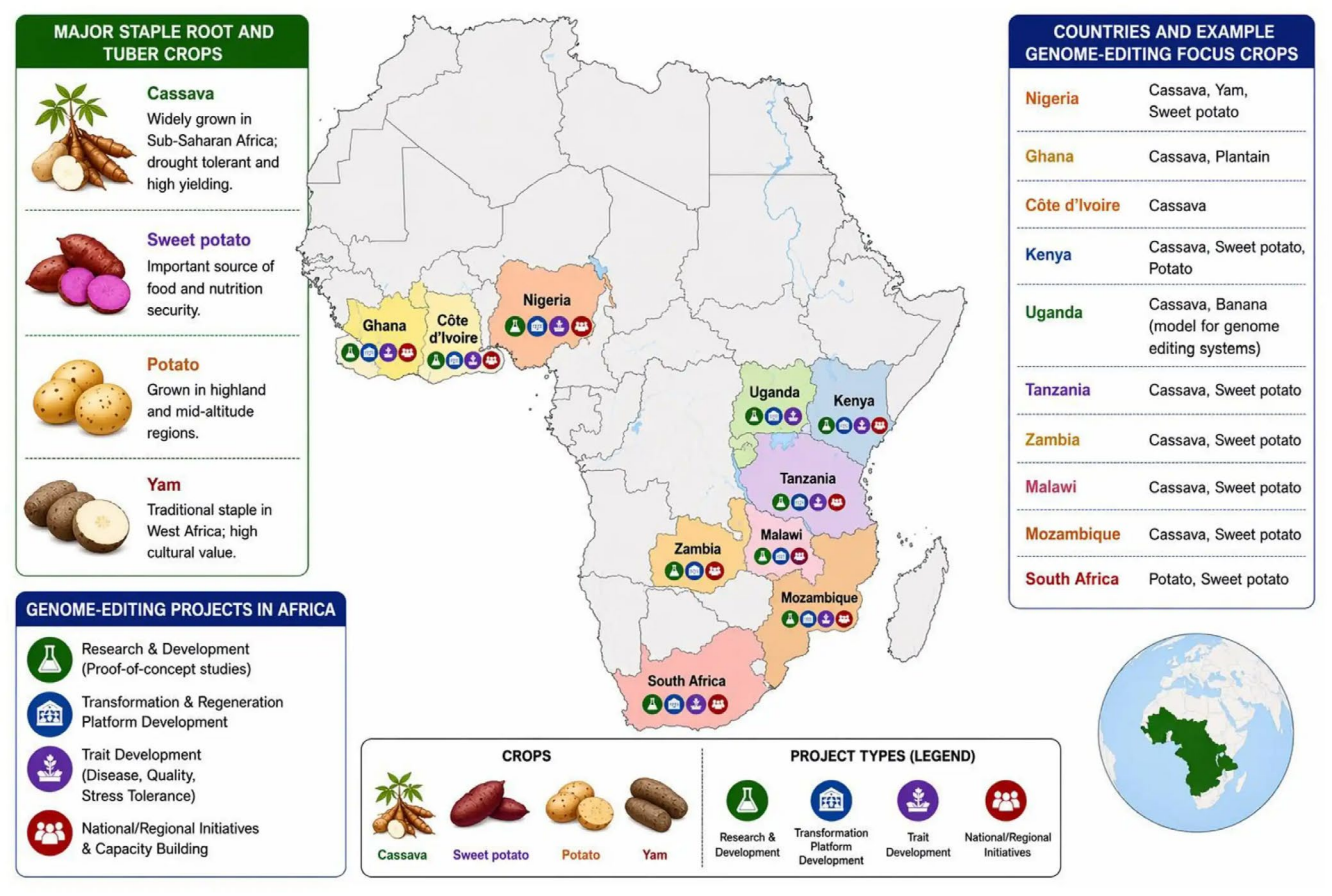
Map of Africa highlighting the major staple food crops discussed in this article, along with countries where genome editing projects are currently being implemented. The data are compiled from published reports of genome editing activities in Africa (e.g., Karembu 2021; Tripathi et al. 2024) (Figure created by the authors using BioRender.com).

This review synthesizes recent progress in applying genome editing to root and tuber crops, which are vital to the livelihoods of billions of people. In particular, CRISPR/Cas9 technology has enabled precise modifications that improve yield potential, nutritional composition, and resistance to biotic stresses (Divya et al. 2024). Since the advent of CRISPR/Cas9 technology, numerous African researchers have leveraged this tool for crop improvement. Genome editing initiatives in Africa have focused on improving staple crops through targeted trait modification, particularly for disease resistance and yield stability under climate stress (Karembu 2021). The countries highlighted in (Figure 1) represent regions where active genome editing projects are currently being implemented and are included here as illustrative examples within a broader global context. This article reviews recent advances in CRISPR/Cas9‐mediated genome editing of root and tuber crops across multiple regions.

## 2 Genome Editing Tools for Root & Tuber Crop Improvement

Genome editing technologies enable precise, sequence‐specific modification of plant genomes and have been increasingly applied to improve key traits in root and tuber crops. Among these, CRISPR/Cas systems are the most widely used due to their efficiency, flexibility, and ease of design. Gene editing has emerged as an important approach for crop improvement, with the capacity to substantially enhance productivity and contribute to global food and energy security (Chen et al. 2024; Gomez et al. 2019; Veillet et al. 2019).

CRISPR/Cas9 is the most widely used genome editing system in plant research and crop improvement, consisting of a Cas9 nuclease and a guide RNA (gRNA) (Tussipkan and Manabayeva 2021). Together, they induce targeted DNA double‐strand breaks (DSBs) (Doudna and Charpentier 2014), which are subsequently repaired by cellular DNA repair pathways. CRISPR/Cas9‐mediated genome editing involves guide RNA‐directed targeting of specific genomic loci, followed by DNA cleavage and repair through endogenous pathways, typically resulting in gene disruption or targeted modification. In root and tuber crops, the efficiency of this process is strongly influenced by transformation and regeneration systems, which remain key technical constraints.

**FIGURE 2:**
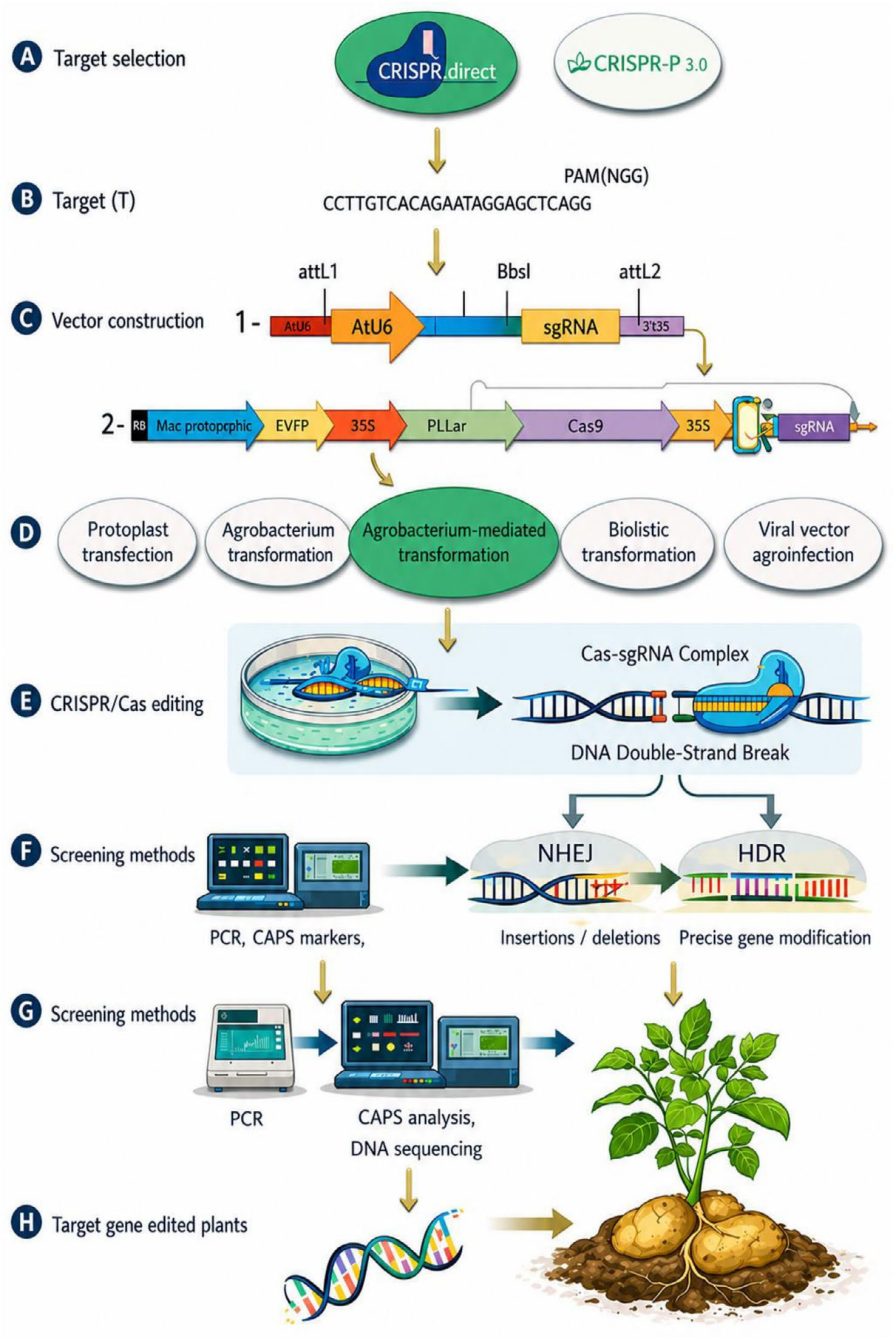
Conceptual workflow of CRISPR/Cas‐mediated genome editing in root and tuber crops, highlighting key steps and crop‐specific constraints, including transformation efficiency, regeneration capacity, and polyploid genome complexity. The workflow is adapted from published studies on plant genome editing (e.g., Chen et al. 2024; Tripathi et al. 2024; Tussipkan and Manabayeva 2021) (Figure created by the authors using BioRender.com).

In CRISPR/Cas‐mediated genome editing, the Cas‐guide RNA complex targets specific DNA sequences and induces site‐specific modifications that are repaired by endogenous cellular pathways, resulting in gene disruption or precise sequence changes. In root and tuber crops, successful editing depends not only on targeting efficiency but also on effective regeneration and validation of edited plants. A conceptual workflow of CRISPR/Cas‐mediated genome editing in root and tuber crops is illustrated in (Figure 2), highlighting key constraints specific to these crops, including genotype‐dependent transformation, regeneration bottlenecks, polyploid genome complexity, and the need to maintain elite clonal traits during editing.

Unlike seed‐propagated crops, genome editing in root and tuber crops is complicated by clonal propagation, high heterozygosity, and polyploidy, which require precise editing across multiple alleles and efficient regeneration systems.

Advances in CRISPR technology have expanded genome editing capabilities through the development of alternative systems such as Cas12 and Cas13. Cas12 enables flexible DNA targeting and has been applied in genome engineering applications requiring efficient and directional DNA modification (Zetsche et al. 2015). In contrast, Cas13 targets RNA, enabling post‐transcriptional regulation and providing a useful strategy for antiviral defense in plants, particularly against RNA viruses (Abudayyeh et al. 2017; Yu et al. 2022).

These CRISPR systems offer complementary functional advantages depending on the application: Cas9 is widely used for gene knockouts, Cas12 variants expand targeting flexibility, and Cas13 facilitates RNA‐level targeting for viral resistance. Together, they provide adaptable tools for addressing diverse biological challenges in root and tuber crops.

In addition to conventional CRISPR/Cas9 editing, several Cas variants and derivative technologies have been developed to enhance precision and expand editing capabilities for crop improvement (Kaniganti et al. 2026). Cas9 remains the most widely used genome editing system and has been successfully applied to improve traits such as disease resistance and starch quality in root and tuber crops. For example, CRISPR/Cas9‐mediated editing of susceptibility genes such as *StDMR6* has enhanced resistance to late blight in potato (Kieu et al. 2021; Karlsson et al. 2024). Similarly, editing of starch biosynthesis genes including *GBSSI* has enabled modification of starch composition by altering amylose biosynthesis pathways. For example, targeted mutagenesis of *GBSSI* in potato resulted in amylose‐free starch, confirming the central role of this enzyme in amylose biosynthesis (Veillet et al. 2019; Wang et al. 2019).

Cas12a (Cpf1) represents an alternative nuclease with distinct PAM recognition and the ability to generate staggered DNA breaks, making it particularly useful for multiplex genome editing and targeted insertions. More recently, compact nucleases such as Cas12f have attracted attention because their smaller size may facilitate more efficient delivery into plant cells, especially in systems where vector capacity is limited.

In contrast to DNA‐targeting nucleases, Cas13 targets RNA molecules and has been used to confer resistance against RNA viruses. For example, CRISPR/Cas13‐mediated targeting of viral RNA has been shown to provide resistance to sweet potato virus disease by degrading viral transcripts (Yu et al. 2022). Beyond nuclease‐based editing, base editors and prime editors allow precise nucleotide substitutions without generating double‐strand breaks. Cytosine base editors have been successfully applied in potato to introduce targeted nucleotide substitutions in genes such as *GBSSI*, enabling fine‐tuning of starch characteristics (Westberg et al. 2023). Similarly, prime editing has demonstrated the potential for precise sequence modification in polyploid crops such as potato, offering opportunities for targeted allele replacement and trait optimization (Perroud et al. 2022).

Each genome editing platform offers distinct functional advantages; however, their effectiveness depends on alignment with target trait biology, with knockout‐based systems (e.g., Cas9) being most effective for susceptibility gene disruption, while base and prime editing are better suited for precise modification of metabolic and regulatory traits. The integration of these complementary tools provides powerful opportunities for improving disease resistance, starch composition, abiotic stress tolerance, plant architecture, and yield in root and tuber crops. A comparison of major CRISPR/Cas systems and their applications in crop improvement is summarized in Table 1.

**TABLE 1:** Comparison of major CRISPR/Cas genome editing systems and their applications in crop trait improvement.

Editing system	Target molecule	Key advantages	Example applications in crops
Cas9	DNA	High efficiency; widely used; suitable for gene knockout and multiplex editing	Disease resistance, starch modification, yield traits
Cas12a (Cpf1)	DNA	T‐rich PAM recognition; staggered DNA cuts; efficient multiplex editing	Trait stacking, targeted gene insertions
Cas12f	DNA	Compact nuclease; easier delivery using viral vectors	Emerging applications in plant genome editing
Cas13	RNA	RNA targeting; effective for viral RNA degradation	Viral disease resistance
Base editors	DNA	Precise nucleotide substitutions without double‐strand breaks	Starch quality improvement, trait fine‐tuning
Prime editors	DNA	Precise sequence modification including insertions or substitutions	Allele engineering and precise trait modification

An emerging alternative to CRISPR/Cas9 in plant genome editing is the Cas‐CLOVER system, which offers enhanced specificity through a dual‐guide RNA targeting mechanism. By requiring simultaneous binding of two guide RNAs, this system reduces off‐target effects compared with conventional CRISPR approaches (Madison et al. 2022; Tripathi et al. 2023). Cas‐CLOVER has been successfully applied in both monocot and dicot plants, demonstrating high editing efficiency and the ability to generate robust gene knockouts (Madison et al. 2022; Tripathi et al. 2023).

Despite these advantages, the application and commercialization of genome‐edited crops remain influenced by regulatory frameworks, which vary across jurisdictions. In some countries, genome‐edited plants lacking foreign DNA are exempt from GMO regulations, whereas in others, including the European Union, they are regulated under existing GMO policies. This regulatory variability has important implications for the deployment of genome editing technologies, particularly in Sub‐Saharan Africa, where evolving regulatory frameworks may be influenced by international policy approaches and can affect technology adoption, research collaboration, and trade (Sánchez 2024; Tachikawa and Matsuo 2024).

CRISPR‐Cas9 is the preferred gene‐editing tool across a wide range of species, including root and tuber crops, owing to its ease of guide RNA design, minimal requirement for protein engineering, cost‐effectiveness, and high efficiency in generating targeted mutations (Saini et al. 2023). However, the risk of off‐target mutations and unintended genomic alterations can raise biosafety and regulatory concerns, particularly regarding the accuracy of edits, potential unintended effects on gene function, and the need for comprehensive molecular characterization prior to approval. In comparison, TALENs provide greater specificity through longer DNA recognition sequences, though they are more labor‐intensive and costly. Zinc finger nucleases (ZFNs), once widely used, have largely been replaced by CRISPR and TALENs due to the simplicity and flexibility of CRISPR‐based systems (Doudna and Charpentier 2014; Chen et al. 2024). The Cas‐CLOVER system offers a high‐precision alternative with minimal off‐target effects, making it particularly suitable for sensitive applications.

Root and tuber crops present significant breeding challenges due to irregular or absent flowering, poor seed set, and complex genetic backgrounds that require extensive backcrossing to achieve homozygosity. Genome editing provides a powerful and increasingly applied approach by enabling precise modifications of target genes, particularly those regulating flowering, thereby facilitating the development of improved crop varieties essential for agricultural sustainability. CRISPR/Cas9 has the potential to revolutionize the production of vegetatively propagated crops by introducing traits such as disease resistance, drought tolerance, and enhanced nutritional quality, ultimately boosting productivity and sustainability (Lakhani et al. 2023). Table 2 presents recent advancements in genome editing of root and tuber crops. Overall, the effectiveness of genome editing in root and tuber crops depends on the alignment between editing strategy and trait biology, highlighting the importance of mechanism‐driven target selection.

**TABLE 2:** Recent advancements in genome editing of root and tuber crops.

Crop	Target gene(s)/locus	Trait/outcome	Editing system	Delivery/regeneration	Year	References
Potato ( *Solanum tuberosum* )	5′ UTR/promoter of Vacuolar Invertase (VInv/StVInv)	Reduced cold‐induced sweetening; lower reducing sugars in cold‐stored tubers	CRISPR/Cas9 (knockout/indel in regulatory region)	Agrobacterium‐mediated transformation; tissue culture regeneration	2024	Shumbe et al. (2024)
Potato ( *S. tuberosum* )	Polyphenol oxidase gene StPPO2 (multiplexed)	Reduced enzymatic browning; improved processing quality	CRISPR/Cas9 (multiplex)	Agrobacterium; tissue culture regeneration	2023	Ly et al. (2023)
Potato ( *S. tuberosum* )	Granule‐bound starch synthase I (StGBSSI)	Waxy (amylose‐free/low) tuber starch; altered starch functionality	Cytidine base editor + CRISPR/Cas9	Protoplast transfection; regeneration	2019	Veillet et al. (2019)
Potato ( *S. tuberosum* )	eIF4E1 (translation initiation factor)	Extended resistance spectrum to PVY strains	CRISPR/Cas9 (knockout)	Agrobacterium; regeneration in cv. Désirée	2022	Lucioli et al. (2022)
Cassava ( *Manihot esculenta* )	Phytoene desaturase (MePDS)	Albino/photobleaching marker; first CRISPR PoC in cassava	CRISPR/Cas9	Agrobacterium; friable embryogenic callus (FEC) regeneration	2017	Odipio et al. (2017)
Cassava ( *M. esculenta* )	nCBP‐1 & nCBP‐2 (eIF4E isoforms)	Reduced severity/incidence of cassava brown streak disease (CBSD)	CRISPR/Cas9 (simultaneous editing)	Agrobacterium; FEC regeneration	2019	Gomez et al. (2019)
Cassava ( *M. esculenta* )	Virus‐enabled expression (VOX) system: CsCMV2‐NC vector (enabler for VIGE/VOX)	In planta protein expression; enabling genome editing delivery strategies in cassava	Virus vector platform (CsCMV‐based)	Mechanical inoculation; systemic infection in cassava	2023	Tuo et al. (2023)
Sweet potato ( *Ipomoea batatas* )	IbGBSSI; IbSBEII	Modified starch quality (high‐amylopectin/altered amylose)	CRISPR/Cas9 (knockout)	Agrobacterium; regeneration of edited lines	2019	Wang et al. (2019)
Sweet potato ( *I. batatas* )	SPCSV RNase3 (viral gene)	Resistance to sweet potato virus disease (SPVD) in transgenic plants	CRISPR/Cas13 (RfxCas13d RNA targeting)	Agrobacterium; transgenic expression of Cas13 and gRNAs	2022	Yu et al. (2022)
Yam (*Dioscorea* spp.)	Phytoene desaturase (PDS)	Established first efficient genome editing system in yam; albino phenotype marker	CRISPR/Cas9	Agrobacterium; embryogenic tissues	2021	Syombua et al. (2021)
Carrot ( *Daucus carota* )	DcPDS and other loci (first demonstration in carrot)	Proof‐of‐concept carrot genome editing; visible albino phenotype	CRISPR/Cas9	Agrobacterium; callus/protoplast workflows	2018	Klimek‐Chodacka et al. (2018)
Carrot ( *D. carota* )	Multiple loci (transgene‐free)	Transgene‐free edited plants via RNP delivery	Cas9 RNP (DNA‐free)	PEG‐mediated protoplast transfection; plant regeneration	2025	Yarra and Krysan (2025)
Radish ( *Raphanus sativus* )	RsGL1a and RsGL1b (GLABRA1 orthologs)	Glabrous (trichome‐less) phenotype; established radish CRISPR pipeline	CRISPR/Cas9	Agrobacterium; tissue culture regeneration	2022	Muto and Matsumoto (2022)
Sugar beet ( *Beta vulgaris* )	Becurtovirus (Beet curly top virus) genome targets (multiplex)	Broad‐spectrum resistance; inhibition of systemic infection	CRISPR/Cas9 (antiviral)	Agrobacterium; transgenic lines	2023	Yıldırım et al. (2023)

## 3 Rationale and Necessity of Genome Editing for Root and Tuber Crop Improvement

Root and tuber crops, including potato ( *Solanum tuberosum* ), cassava ( *Manihot esculenta* ), sweet potato ( *Ipomoea batatas* ), and yam (*Dioscorea* spp.), are cornerstone staples for billions of people globally, particularly in Sub‐Saharan Africa, where they provide essential carbohydrates, micronutrients, and dietary energy. Despite their global importance, genetic improvement of root and tuber crops through conventional breeding remains challenging due to several biological and agronomic constraints. Many root and tuber crops are polyploid (e.g., tetraploid potato and hexaploid sweet potato) and highly heterozygous, complicating the identification, selection, and fixation of desirable alleles (Lakhani et al. 2023; Divya et al. 2024). In addition, their predominant vegetative propagation limits meiotic recombination and prolongs breeding cycles, making it difficult to introduce new traits without disrupting elite cultivar characteristics, such as yield stability, tuber quality, processing attributes, and adaptation to local agroecological conditions. These limitations are further exacerbated by susceptibility to major pathogens, including *Phytophthora infestans* (late blight in potato) and cassava mosaic virus, as well as increasing abiotic stresses such as drought and heat associated with climate change.

Genome editing technologies, particularly CRISPR/Cas‐based systems, provide a well‐established approach for precise, site‐specific modification of target genes. Compared with conventional breeding, genome editing enables direct manipulation of causal genes, including susceptibility factors and metabolic enzymes, while maintaining elite genetic backgrounds (Kieu et al. 2021; Veillet et al. 2019). The effectiveness of this approach depends on the biological role of the targeted gene. For example, editing translation initiation factors such as *eIF4E* in cassava confers resistance to potyviruses by disrupting the interaction between viral VPg proteins and the host translation machinery, thereby preventing viral RNA translation and replication (Bastet et al. 2019; Gomez et al. 2019). Similarly, targeted modification of the *GBSSI* gene alters starch biosynthesis by reducing amylose content through disruption of ADP‐glucose‐mediated glucan chain elongation. This results in amylose‐free or low‐amylose (“waxy”) starch with improved industrial properties, including enhanced paste clarity, modified viscosity, and better digestibility (Veillet et al. 2019; Wang et al. 2019).

In addition to quality traits, genome editing enables precise engineering of agronomic traits such as herbicide tolerance. Edits in genes such as *ALS1* and *EPSPS* typically involve targeted point mutations that alter herbicide‐binding sites while retaining enzyme function, thereby conferring resistance without compromising essential metabolic pathways. Similarly, targeted modification of resistance (*R*) genes or susceptibility genes in potato has enhanced resistance to *Phytophthora infestans* while maintaining yield and tuber quality (Kieu et al. 2021). Collectively, these examples demonstrate that genome editing enables mechanism‐driven trait improvement, offering a more predictable and efficient route to developing resilient, high‐performing root and tuber crops varieties. As such, genome editing is emerging as an indispensable tool for addressing the urgent need to enhance productivity, quality, and climate resilience in root and tuber crops (Tussipkan and Manabayeva 2021; Tripathi et al. 2022).

## 4 Recent Advances in Genome Editing of Root and Tuber Crops

Recent advances in genome editing have enabled mechanism‐driven improvement of root and tuber crops, including cassava, potato, sweet potato, and yam, key staples essential for global food security and livelihoods (Saini et al. 2025) (Table 3). In cassava, targeted knockout of *CYP79D1* and *CYP79D2* reduces cyanogenic glucoside accumulation by disrupting the initial step of linamarin biosynthesis, in which these cytochrome P450 enzymes convert valine into cyanogenic intermediates. Blocking this pathway prevents downstream release of toxic hydrogen cyanide, thereby improving food safety and processing quality (Gomez et al. 2023). In addition, editing of susceptibility‐associated genes such as *MeSWEET10a* enhances resistance to bacterial blight by limiting pathogen‐induced sugar efflux required for bacterial growth and virulence (Wang et al. 2022; Wang et al. 2023).

**TABLE 3:** Recent advances and applications of genome editing in root and tuber crops.

Crop	Gene target/system	Mechanism/trait outcome	References
Cassava	*CYP79D1/D2*	Disruption of cyanogenic glucoside biosynthesis by blocking valine → oxime conversion, reducing linamarin and cyanide release	Gomez et al. (2023)
Cassava	*MeSWEET10a* promoter	Reduced pathogen‐induced sugar efflux, limiting bacterial growth and conferring blight resistance	Wang et al. (2022); Mukami et al. (2024)
Cassava	nCBP‐1/nCBP‐2	Disruption of viral VPg–eIF4E interaction, inhibiting viral RNA translation and CBSD progression	Gomez et al. (2019)
Cassava	CsCMV‐based viral vector	In planta delivery of editing components, enabling transient expression and reducing tissue culture dependency	Tuo et al. (2023)
Potato	*StDMR6‐1*	Loss of susceptibility gene enhances resistance via activation of salicylic acid‐mediated defense pathways	Kieu et al. (2021)
Potato	*St16DOX*/*StPM1*	Modification of metabolic/susceptibility pathways reduces pathogen compatibility and improves resistance	Bi et al. (2024)
Potato	*GBSSI*	Disruption of amylose biosynthesis via ADP‐glucose incorporation, producing amylose‐free starch	Veillet et al. (2019)
Potato	*StPPO2* (multiplex)	Reduced enzymatic browning through suppression of polyphenol oxidase activity	Ly et al. (2023)
Sweet potato	*IbGBSSI*	Reduced amylose synthesis, improving starch digestibility and industrial processing quality	Wang et al. (2019)
Sweet potato	*IbSBEII*	Altered branching in amylopectin, modifying starch structure and functional properties	Wang et al. (2019)
Sweet potato	SPCSV RNase3 (Cas13)	RNA targeting of viral genome, conferring resistance to sweet potato virus disease	Yu et al. (2022)
Yam	*DrPDS*	Disruption of carotenoid biosynthesis (albino phenotype), enabling functional validation of genome editing	Syombua et al. (2021); Agre et al. (2026)

In potato, genome editing has improved both disease resistance and quality traits through targeted modification of susceptibility genes and metabolic pathways. For example, disruption of susceptibility‐related genes enhances resistance to *Phytophthora infestans* by reducing host compatibility with the pathogen, while maintaining agronomic performance (Kieu et al. 2021; Bi et al. 2024). Editing of starch biosynthesis genes such as *GBSSI* alters amylose content by impairing ADP‐glucose‐mediated glucan chain elongation, resulting in modified starch functionality relevant for food processing and industrial applications (Veillet et al. 2019).

In sweet potato, editing of *IbGBSSI* and *IbSBEII* enables precise modulation of starch composition by altering the balance between amylose and amylopectin synthesis, thereby improving textural and nutritional properties (Wang et al. 2019). In yam, CRISPR/Cas9‐mediated editing of *DrPDS* has established a functional genome editing platform, enabling gene validation and providing a foundation for future trait improvement in this previously recalcitrant crop (Syombua et al. 2021).

These advances are supported by improvements in enabling technologies. Viral vector‐mediated delivery systems, such as cassava mosaic virus‐based platforms, enable in planta delivery of genome editing components without stable transformation, thereby reducing reliance on tissue culture and improving editing efficiency (Tuo et al. 2023). Multiplex CRISPR strategies, which utilize multiple guide RNAs to simultaneously target several genes, allow coordinated modification of complex traits controlled by gene networks. In addition, AI‐assisted functional genomics resources integrate genomic, transcriptomic, and phenotypic datasets to identify key regulatory genes and prioritize editing targets, thereby accelerating trait discovery and genome editing design (Li et al. 2026; Zhou et al. 2024).

Collectively, these studies demonstrate a transition from single‐gene modification toward integrated, mechanism‐based genome editing strategies, enabling more precise and efficient improvement of complex traits in root and tuber crops.

## 5 Accelerating Genetic Gains in Root and Tuber Crops Through Advanced Genome Editing

Genome editing tools, particularly CRISPR/Cas systems, have contributed to accelerating genetic gains in root and tuber crops by enabling targeted improvements in traits such as disease resistance, post‐harvest quality, and starch composition (Kieu et al. 2021; Gomez et al. 2019; Veillet et al. 2019; Wang et al. 2019), with broader trends summarized in recent reviews (Tripathi et al. 2024; Chen et al. 2024). Genomic resources for staple species including cassava, potato, sweet potato, and yams have expanded significantly, facilitating efficient genome editing and trait enhancement in these complex, vegetatively propagated crops (Divya et al. 2024). In potatoes, CRISPR/Cas9 editing of *GBSS1*, *SBE1*, and *SBE2* genes has enabled production of transgene‐free lines with modified starch composition by altering the balance between amylose and amylopectin biosynthesis; specifically, disruption of GBSS1 reduces amylose formation due to impaired ADP‐glucose‐mediated chain elongation, while SBE mutations affect branching architecture of amylopectin (Andersson et al. 2017, 2018; Toinga‐Villafuerte et al. 2022; Kusano et al. 2018). Further CRISPR innovations in potato include knocking out St16DOX to eliminate toxic glycoalkaloids and StPM1, enhancing resistance to *Phytophthora* pathogens without compromising growth (Bi et al. 2024; Nahirñak et al. 2022). For instance, CRISPR/Cas9‐mediated editing of *StDMR6‐1* significantly enhanced resistance to *Phytophthora infestans* without yield penalty, demonstrating the effectiveness of susceptibility gene targeting (Kieu et al. 2021; Karlsson et al. 2024). Advancements in cassava include CRISPR‐mediated gene tagging via homology‐directed repair and epigenome editing strategies to improve bacterial blight resistance (Veley et al. 2021, 2023). Simultaneous editing of *nCBP‐1* and *nCBP‐2* in cassava reduced cassava brown streak disease severity by disrupting host factors required for viral RNA translation (Gomez et al. 2019). In addition, CRISPR/Cas9‐mediated targeted mutagenesis of MeF6′H genes has been shown to significantly reduce post‐harvest physiological deterioration (PPD) in cassava storage roots, thereby improving shelf life and post‐harvest quality (Mukami et al. 2024). In sweet potato, editing of starch‐biosynthetic genes (IbGBSSI, IbSBEII) has demonstrated precise modulation of starch quality, while early CRISPR efforts in yam targeting visual marker genes like DrPDS lay groundwork for future trait engineering (Wang et al. 2019; Divya et al. 2024). Together, these breakthroughs illustrate how genome editing is transforming plant breeding in root and tuber crops and paving the way for resilient, high‐performing varieties across diverse agricultural landscapes. Despite these advances, translation remains constrained by transformation efficiency, polyploid genome complexity, and regeneration bottlenecks as discussed in next section.

### 5.1 Cross‐Crop Comparative Insights

**FIGURE 3:**
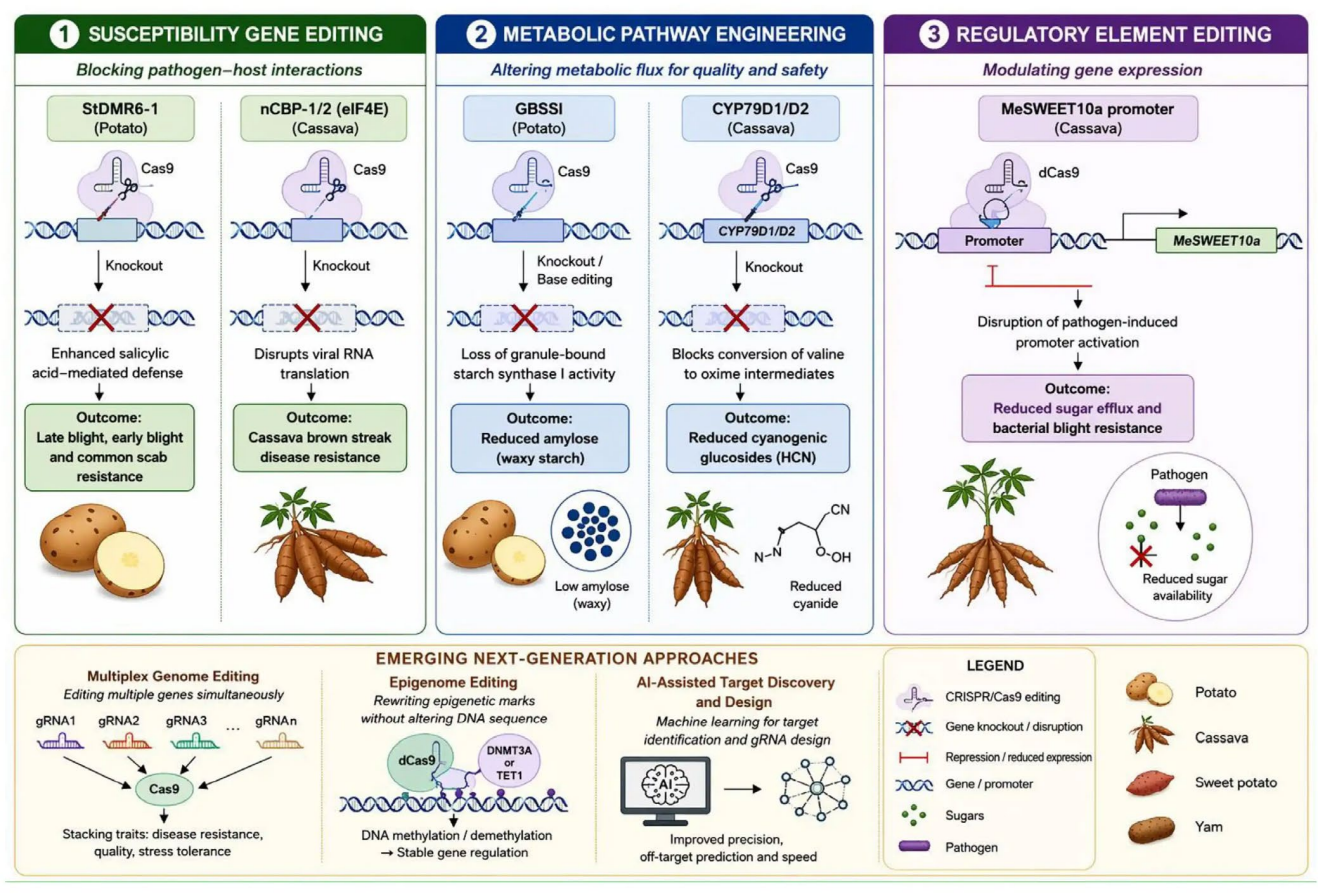
Mechanistic framework of genome editing strategies in root and tuber crops. The diagram illustrates three core editing strategies: (i) susceptibility gene disruption (e.g., DMR6, eIF4E) to enhance disease resistance by blocking pathogen‐host interactions, (ii) metabolic pathway engineering (e.g., GBSSI, CYP79D) to modify starch composition and reduce toxic metabolites, and (iii) regulatory element editing (e.g., MeSWEET10a promoter) to fine‐tune gene expression. Emerging approaches including multiplex genome editing, epigenome editing, and AI‐assisted target discovery are highlighted as next‐generation strategies for engineering complex traits in polyploid crops. The concepts illustrated are based on published studies on genome editing applications and strategies in root and tuber crops (e.g., Kieu et al. 2021; Veillet et al. 2019; Chen et al. 2024; Tripathi et al. 2024) (Figure created by the authors using BioRender.com).

Despite the diversity of applications, genome editing efforts in root and tuber crops broadly converge on three principal mechanistic strategies. First, disruption of susceptibility genes (e.g., *DMR6*, *eIF4E*) has been widely employed to achieve durable resistance against pathogens by impairing host factors required for pathogen establishment and replication. Second, metabolic pathway rewiring (e.g., *GBSSI*, *CYP79D*) enables targeted modification of key biosynthetic processes, leading to improved quality traits such as starch composition and reduced accumulation of toxic metabolites. Third, regulatory element editing, including promoter modifications (e.g., *MeSWEET10a*), facilitates precise spatiotemporal control of gene expression, particularly in response to biotic stress signals. A unified mechanistic framework summarizing major genome editing strategies across root and tuber crops is presented in (Figure 3).

However, cross‐species comparisons reveal a critical limitation in current approaches. Most genome editing studies in root and tuber crops remain focused on single‐gene targets, whereas key agronomic traits such as yield stability, abiotic stress tolerance, and resource‐use efficiency are governed by complex, polygenic regulatory networks. Most successful genome editing applications in root and tuber crops have focused on single‐gene targets, such as susceptibility genes and key metabolic enzymes, as demonstrated in studies on *StDMR6‐1*, *nCBP*, and *GBSSI* (Kieu et al. 2021; Gomez et al. 2019; Veillet et al. 2019). This gap reflects a mismatch between the predominantly single‐gene focus of current genome editing applications and the complex, polygenic architecture of target traits, with broader perspectives discussed in recent reviews (Chen et al. 2024; Tripathi et al. 2024).

Addressing this limitation will require a strategic transition toward multiplex and network‐level genome editing, enabling simultaneous manipulation of multiple genes and regulatory circuits. Emerging advances in CRISPR multiplexing, epigenome editing, and AI‐assisted functional genomics are beginning to support this shift, offering new opportunities to decode and engineer complex trait architectures in polyploid and clonally propagated crops (Li et al. 2026; Chen et al. 2024). Such integrative approaches will be essential for unlocking the full potential of genome editing in root and tuber crops improvement and for bridging the gap between laboratory‐scale innovations and field‐level performance.

Collectively, these studies demonstrate that genome editing strategies targeting susceptibility genes and key metabolic pathways consistently yield more predictable and durable trait improvements compared to conventional breeding approaches, due to their ability to directly modify causal genetic determinants. Despite these advances, translation remains constrained by transformation efficiency, polyploid genome complexity, and regeneration bottlenecks as discussed in the next section. These examples illustrate a unifying principle in root and tuber crops genome editing: targeted manipulation of host susceptibility factors, metabolic enzymes, and biosynthetic pathway genes enables predictable trait modification through well‐defined molecular mechanisms.

Analysis of primary studies across root and tuber crops indicates that most successful genome editing applications target either susceptibility genes or key metabolic enzymes. However, relatively few studies address polygenic traits such as yield or abiotic stress tolerance, highlighting a major gap in current research and emphasizing the need for multiplex and network‐level editing approaches. A comparative summary of key genome editing targets, their molecular mechanisms, and resulting trait outcomes across root and tuber crops is presented in Table 4.

**TABLE 4:** Mechanistic comparison of genome editing targets in root and tuber crops.

Crop	Gene target	Biological function	Editing strategy	Mechanistic outcome	Trait improvement	References
Potato	*StDMR6‐1*	Susceptibility gene (SA metabolism)	Knockout	Activates defense pathways	Late blight resistance	Kieu et al. (2021); Karlsson et al. (2024)
Cassava	*nCBP‐1/2* (eIF4E)	Translation initiation factor	Knockout	Disrupts viral RNA translation	CBSD resistance	Gomez et al. (2019)
Cassava	*CYP79D1/D2*	Cyanogenic glucoside biosynthesis	Knockout	Blocks valine → oxime conversion	Reduced cyanide	Gomez et al. (2023)
Potato	*GBSSI*	Starch biosynthesis enzyme	Knockout/Base editing	Disrupts amylose formation	Waxy starch	Veillet et al. (2019); Wang et al. (2019)
Cassava	*MeSWEET10a*	Sugar transporter	Promoter editing	Limits pathogen sugar access	Bacterial blight resistance	Wang et al. (2022)
Sweet potato	*IbSBEII*	Starch branching enzyme	Knockout	Alters amylopectin structure	Improved starch quality	Wang et al. (2019)
Potato	*StPPO2*	Polyphenol oxidase	Knockout	Reduces enzymatic browning	Processing quality	Ly et al. (2023)
Root and tuber crops	—	Integrated genomic‐assisted breeding	Genome editing + breeding integration	Enhances trait selection and deployment efficiency	Improved breeding pipelines	Agre et al. (2026)

## 6 Current Limitations and Future Directions

Root and tuber crops present distinct biological and technical challenges for genome editing, particularly due to gene redundancy and pleiotropy, where multiple genes perform overlapping functions or a single gene influences multiple traits. In such cases, editing a single gene may result in limited phenotypic effects or unintended trade‐offs in growth, yield, or quality, necessitating simultaneous targeting of multiple gene copies or regulatory pathways (Chincinska et al. 2023; Chen et al. 2024).

A major practical constraint remains the transformation and regeneration process, which is highly genotype‐dependent in most root and tuber crops. Many farmer‐preferred cultivars exhibit low transformation efficiency, and tissue culture‐based regeneration often introduces somaclonal variation, referring to unintended genetic or epigenetic changes arising during in vitro culture that can lead to phenotypic instability. Previous studies have reported important progress in addressing these challenges. For example, Elegba et al. (2021) demonstrated successful transformation and regeneration of a farmer‐preferred Ghanaian cassava cultivar through optimization of *Agrobacterium*‐mediated infection parameters and regeneration media. Similarly, Segatto et al. (2022) reported improved transformation efficiency in cassava by refining explant selection, infection conditions, and culture environments. However, these advances remain protocol‐specific and genotype‐dependent, requiring extensive optimization and continued reliance on tissue culture systems, which still pose risks of somaclonal variation and chimerism. These findings reinforce the need for genotype‐independent transformation systems and in planta editing approaches to enable broader application across diverse cultivars.

Emerging strategies such as DNA‐free ribonucleoprotein (RNP) delivery and in planta transformation systems aim to overcome these limitations by reducing reliance on tissue culture and minimizing unintended genomic alterations, although their efficiency remains variable across species (Ma et al. 2024; Shen et al. 2024).

Advances in editing technologies, including base editors and prime editors, offer improved precision by enabling targeted nucleotide substitutions without inducing double‐strand breaks. Base editors mediate direct nucleotide conversions (e.g., C → T or A → G), while prime editors enable programmable insertions, deletions, or substitutions using reverse transcriptase guided repair. However, their application in polyploid RTC genomes remains constrained by variable efficiency and challenges in editing multiple alleles simultaneously (Perroud et al. 2022; Chen et al. 2024).

Another major challenge is the accurate genotyping of edited plants, particularly in polyploid species. Techniques such as allele phasing, which distinguish and characterize individual alleles within a genome, are essential for confirming precise edits and avoiding chimeric or partially edited plants. These approaches require high‐throughput sequencing and advanced bioinformatics tools, increasing the complexity and cost of validation (Chen et al. 2024).

Bridging the gap between laboratory success and field application remains a critical bottleneck. Traits such as yield, stress tolerance, and post‐harvest performance must be validated across diverse environments. Participatory field trials, involving collaboration with farmers and local stakeholders, have proven effective in crops such as cassava for evaluating trait performance under real‐world conditions and improving adoption (Sánchez 2024).

To accelerate deployment, there is a need for open‐access genome editing toolkits and platforms, including publicly available CRISPR vector systems, genome databases, and AI‐assisted functional genomics resources that integrate multi‐omics data to identify key regulatory targets and guide editing strategies. Initiatives such as open CRISPR libraries and crop‐specific genomic databases are helping democratize access to genome editing technologies, particularly for public‐sector breeding programs (Zhou et al. 2024; Li et al. 2026).

Finally, harmonization of regulatory frameworks and clearer guidelines for DNA‐free genome editing will be essential for global adoption. Coordinated efforts among researchers, policymakers, and stakeholders are required to ensure safe, equitable, and efficient deployment of genome editing technologies in root and tuber crops (Sánchez 2024; Tachikawa and Matsuo 2024). Continued improvements in transformation efficiency, delivery systems, and regeneration protocols will be critical for translating genome editing advances from laboratory research to field‐level crop improvement.

### 6.1 Emerging Conceptual Advances

Future progress in genome editing of root and tuber crops will depend on a paradigm shift from single‐gene modification toward systems‐level genome engineering (Kaniganti et al. 2026). While early applications have primarily focused on targeted gene knockouts, recent advances in CRISPR‐based technologies highlight the potential for coordinated manipulation of multiple loci, enabling more precise and scalable trait engineering in complex crop systems (Chen et al. 2024; Tripathi et al. 2024).

One of the most promising directions is multiplex genome editing, which allows simultaneous modification of multiple genes or regulatory elements within a pathway. This approach is particularly important for improving complex traits such as tuber development, yield stability, and abiotic stress tolerance, which involve coordinated regulation of multiple genes and pathways. Multiplex genome editing, enabled by the use of multiple guide RNAs targeting several loci simultaneously, has been demonstrated in crop systems to facilitate coordinated trait modification (Wang et al. 2017; Kieu et al. 2021). Advances in guide RNA design, delivery systems, and editing efficiency are further supporting the practical implementation of multiplex strategies in vegetatively propagated crops, as highlighted in recent studies and reviews (Chen et al. 2024; Tripathi et al. 2024).

In parallel, precision editing technologies, including base editing and prime editing, are expanding the scope of genome engineering by enabling targeted nucleotide substitutions without introducing double‐strand breaks. These tools allow fine‐tuning of gene function and regulatory sequences, offering greater control over trait expression while minimizing unintended genomic alterations (Chen et al. 2024; Perroud et al. 2022). Such precision is particularly advantageous in clonally propagated RTCs, where maintaining elite genetic backgrounds is essential.

Another emerging direction is the integration of AI‐guided target discovery and multi‐omics approaches, which combine genomic, transcriptomic, and metabolomic datasets to identify key regulatory genes and networks. These data‐driven frameworks improve target selection accuracy and enable more rational design of genome editing strategies, thereby accelerating the development of improved crop varieties (Li et al. 2026; Chen et al. 2024).

Furthermore, allele‐specific editing in polyploid genomes represents a critical advancement for root and tuber crops such as potato and sweet potato, where multiple gene copies and high heterozygosity complicate conventional editing approaches. Targeted modification of specific alleles enables fine‐tuning of gene dosage and function without complete gene knockout, thereby preserving beneficial allelic variation while optimizing trait performance (Tripathi et al. 2024).

Collectively, these emerging innovations signal a transition from conventional “gene editing” toward “genome design”, where coordinated manipulation of genes, regulatory elements, and biological networks enables rational engineering of complex traits. This conceptual shift will be essential for fully harnessing genome editing technologies to improve productivity, resilience, and quality in root and tuber crops under changing climatic conditions (Chen et al. 2024; Tripathi et al. 2024).

### 6.2 Future Research Priorities

To fully realize the potential of genome editing in root and tuber crops, several key research priorities must be addressed. A primary focus should be the development of genotype‐independent transformation systems, which would enable efficient editing across diverse cultivars, including farmer‐preferred and recalcitrant genotypes. Current transformation protocols remain highly genotype‐specific and continue to limit the scalability and practical deployment of genome editing technologies in many root and tuber crops (Tripathi et al. 2024; Chen et al. 2024).

Another critical priority is the optimization of precision editing technologies, particularly prime editing, in polyploid crops. While prime editing offers the ability to introduce precise nucleotide substitutions and small insertions without double‐strand breaks, its efficiency in complex, polyploid genomes such as potato and sweet potato remains relatively low and requires further refinement (Perroud et al. 2022; Chen et al. 2024).

The integration of speed breeding with genome editing represents an additional opportunity to accelerate crop improvement. By shortening generation cycles and enabling rapid fixation and evaluation of edited alleles, this combined approach has the potential to significantly reduce the time required for developing improved root and tuber crops varieties, particularly in crops with long breeding cycles (Tripathi et al. 2024).

Advances in field‐scale phenomics, particularly for underground traits, are also essential. High‐throughput and non‐destructive phenotyping tools for root architecture, tuber development, and post‐harvest traits remain underdeveloped, limiting the ability to evaluate genome‐edited lines under realistic field conditions. Improved phenotyping platforms will be critical for linking genotype to phenotype and validating trait performance across diverse environments (Chen et al. 2024).

In addition, the development of transgene‐free genome editing pipelines is crucial for improving regulatory acceptance and facilitating commercialization. DNA‐free editing approaches, such as ribonucleoprotein (RNP)‐mediated delivery and transient expression systems, can reduce regulatory barriers and enhance public acceptance, particularly in regions with strict GMO policies (Tripathi et al. 2024; Sánchez 2024).

Importantly, future research must extend beyond laboratory and greenhouse studies to include multi‐environment field validation and socio‐economic impact assessment. Many genome editing studies in root and tuber crops remain limited to controlled conditions, with insufficient evaluation of yield stability, stress resilience, and farmer adoption under real‐world scenarios. Addressing these gaps will be essential for translating genome editing innovations into sustainable agricultural outcomes and ensuring their relevance to smallholder farming systems (Sánchez 2024; Friedrichs et al. 2022).

## 7 Regulatory Complexities in Genome Editing

Regulatory frameworks governing genome editing in root and tuber crops vary substantially across countries, creating challenges for research, commercialization, and international trade. In some countries, such as the United States, Argentina, and Japan, genome‐edited crops that do not contain foreign DNA are often exempt from strict genetically modified organism (GMO) regulations. In contrast, the European Union regulates most genome‐edited crops under existing GMO legislation, subjecting them to extensive risk assessment and approval processes (Tachikawa and Matsuo 2024; Sánchez 2024). This lack of regulatory harmonization creates uncertainty for developers and limits the global deployment of genome editing technologies.

Root and tuber crops present additional regulatory challenges due to their biological characteristics, including vegetative propagation, polyploid genomes, and high genetic diversity, which complicate molecular characterization and detection of edited alleles. These complexities increase the burden of biosafety assessment, particularly in confirming the absence of unintended edits or off‐target effects (Chen et al. 2024).

Public perception also plays a critical role in the adoption of genome‐edited crops. Studies indicate that acceptance varies depending on perceived benefits, transparency, and trust in regulatory institutions. For example, genome‐edited crops developed for consumer or nutritional benefits tend to receive higher acceptance than those perceived as primarily benefiting producers (Spök et al. 2022; Sánchez 2024). In many developing regions, limited awareness and communication gaps further influence adoption.

Infrastructure and capacity constraints remain significant barriers in many root and tuber crop producing countries. These include limited regulatory expertise, lack of standardized detection systems, and insufficient laboratory facilities for molecular characterization and biosafety evaluation. Such gaps can delay approval processes and restrict the ability of national programs to evaluate and deploy genome‐edited crops effectively (Tachikawa and Matsuo 2024).

To address these challenges, more specific and coordinated actions are required. These include: (i) development of harmonized, science‐based regulatory frameworks that distinguish between transgenic and DNA‐free genome editing approaches; (ii) establishment of regional regulatory hubs and shared biosafety infrastructure to support capacity‐limited countries; (iii) implementation of transparent communication strategies and stakeholder engagement programs to improve public trust; and (iv) promotion of open‐access licensing models and public‐sector breeding initiatives to ensure equitable access to genome editing technologies.

Collectively, these measures will be essential to facilitate the responsible and equitable deployment of genome editing in root and tuber crops, ensuring that technological advances translate into tangible benefits for farmers and consumers worldwide.

## 8 Conclusion

Genome editing has emerged as a powerful tool for improving root and tuber crops by enabling precise modification of genes controlling disease resistance, metabolic pathways, and quality traits. Across crop systems, successful applications converge on three main mechanistic strategies: disruption of susceptibility genes to enhance disease resistance, modification of metabolic pathways to improve quality traits such as starch composition and cyanogenic content, and editing of regulatory elements to fine‐tune gene expression. These approaches have demonstrated clear advantages over conventional breeding by allowing direct improvement of elite cultivars without extensive backcrossing.

Despite these advances, several critical gaps remain. Most genome editing studies in root and tuber crops focus on single‐gene targets, whereas key agronomic traits such as yield, stress tolerance, and storage stability are governed by complex, polygenic networks. In addition, limitations in transformation efficiency, regeneration capacity, and precise editing in polyploid genomes continue to constrain broader application. The lack of robust field‐level validation and socio‐economic assessment further limits translation from laboratory research to real‐world deployment.

Addressing these challenges will require a shift toward multiplex and systems‐level genome engineering, enabling coordinated modification of gene networks underlying complex traits. Future research should prioritize (i) development of genotype‐independent transformation systems, (ii) optimization of precision editing tools such as base and prime editors in polyploid crops, (iii) integration of genome editing with speed breeding and high‐throughput phenomics, and (iv) implementation of multi‐environment field trials to validate performance and stability. In parallel, strengthening regulatory clarity, improving public engagement, and expanding access to genome editing technologies through open platforms will be essential for widespread adoption.

Collectively, these advances signal a transition from gene editing toward genome design, where coordinated and knowledge‐driven manipulation of genetic networks enables sustainable and resilient improvement of root and tuber crops in the face of global food security challenges.

## Funding

The authors have nothing to report.

## Disclosure

The authors have nothing to report.

## Conflicts of Interest

The authors declare no conflicts of interest.

## Data Availability

The authors have nothing to report.
